# Single-Metal Hybrid Micromotor

**DOI:** 10.3389/fbioe.2022.844328

**Published:** 2022-02-14

**Authors:** Dajian Li, Yuhong Zheng, Zhanxiang Zhang, Qi Zhang, Xiaoying Huang, Renfeng Dong, Yuepeng Cai, Lin Wang

**Affiliations:** ^1^ School of Chemistry, South China Normal University, Guangzhou, China; ^2^ State Key Laboratory of Robotics and System, Harbin Institute of Technology, Harbin, China

**Keywords:** micromotor, hybrid, catalysis, ultrasound, group motion

## Abstract

Multimode stimuli-regulated propulsions are extremely useful for artificial micro-/nanomotors in performing specialized tasks in different microscopic environments. However, it is still a great challenge to develop a simple and efficient micro/nanosystem which can operate in complicated environments, either with fuel or without fuel. Here, we report a novel hybrid micromotor which only needs one metal with a special structure: micro-spherical shell with a hole. Since we attractively combine the inherently catalytic properties of Pt for chemical propulsion with a designed concave structure for acoustic propulsion, the micromotors can not only move rapidly in H_2_O_2_ fueled environment due to the chemical reaction between Pt and H_2_O_2_ but also can exhibit excellent acoustic propulsion in a fuel-free environment due to the non-uniform stress caused by ultrasound. In addition, the attractive group motion behavior of the motors, including aggregation, group migration, and dispersion, is easily realized by acoustic field regulation. The brand-new single-metal hybrid micromotors with a dual driving mode, flexible propulsion regulation, and efficient group motion regulation, which are essential for making micro-/nanomotors compatible with different surrounding environments, are expected to advance the field of artificial nanomachines.

## Introduction

Micro-/nanomotors, which can exhibit autonomous self-propulsion by harnessing energy and perform specialized tasks in microscopic environments, have attracted intensive study across many areas of science (Mallouk and Sen, 2009; Abbott et al., 2011; Mei et al., 2011; Wang, 2013; Wang and Pumera, 2015; Ortiz–Rivera et al., 2018; Patiño et al., 2018; Ren et al., 2018; Wang et al., 2018; Khezri and Pumera, 2019). Since the first reported Pt/Au nanorod nanomotors (Paxton et al., 2004), the development of micro-/nanomotors has advanced rapidly in terms of preparation, performance, and practical application. Until now, a variety of micro-/nanomotors have been developed with unique morphologies to utilize different propulsion mechanisms, such as metallic or non-metallic nanowires (Paxton et al., 2004; Wang et al., 2017), Janus microspherical motors (Ebbens and Gregory, 2018; Pourrahimi and Pumera, 2018; Lin et al., 2021), tubular micromotors (Xu et al., 2018; Zha et al., 2018), and helical swimmers (Peyer et al., 2013; Mandal et al., 2018). Due to their excellent controllable motion performance, efficient direction control, and abundant functions, these micro-/nanomotors have been successfully applied for a variety of fields ranging from biological applications (Gao et al., 2021) to environment remediations (Zarei and Zarei, 2018), such as cancer cell capture and isolation (Gao et al., 2017), drug delivery (Medina–Sánchez et al., 2018), sensing applications (Zhang et al., 2019), and water purification (Pumera, 2018).

According to the driven modes, artificial micro-/nanomotors can be classified into chemically driven or physically driven, which correspond to fuel-powered or fuel-free micro-/nanomotors, respectively. Each kind of these motors has their special advantages. For chemically driven micro-/nanomotors, they can harvest the energies directly from the surrounding solutions which are economic, easily operated, and efficient. For physically driven micro-/nanomotors, their propulsions can be flexibly and precisely controlled, including acceleration, deceleration, and stop and go capability, by tuning the parameters of the corresponding devices which can generate physical stimuli, such as light (Dong et al., 2018), ultrasound (Lu et al., 2019), electricity (Bouffier et al., 2016), or magnetism (Li et al., 2016; Li et al., 2018; Lin et al., 2018; Ji et al., 2021). Using different fields to power one artificial micro-/nanomotor offers considerable promise for designing multimodal adaptive micro-/nanovehicles that reconfigure their operation on demand based on changing conditions. However, combining different driven modes into a single device represents a nanoengineering challenge in view of the different requirements of the external stimuli. It is thus critical that the design and fabrication of the multimode micro-/nanomotor couples fuel-powered and fuel-free propulsions in a single micro-/nanomotor which can adapt to complicated conditions. Accordingly, hybrid micro-/nanomotors have been developed (Li et al., 2016). For example, Wei et al. reported a catalytically/magnetically powered adaptive nanowire swimmer: Pt–Au–Ag_flex_–Ni nanowire motor (Gao et al., 2011). The motor has a Pt–Au nanorod head for catalytic propulsion and a Ni tail connected by a flexible Ag nanowire for magnetic propulsion. Li et al. reported a magneto-acoustic hybrid nanomotor, which has a gold nanorod for acoustic propulsion and a Pd/Ni helical nanowire for magnetic propulsion (Li et al., 2015). It is clear to see that such hybrid micromotors require two functional components which correspond to the two propulsion modes. These conventional design strategies for construction of dual-driven micro-/nanomotors involve complicated fabrication processes and require multiple materials or complex structures, driving increased costs. Therefore, combining multiple driven modes to a single micromotor by a simple strategy is essential to design advanced micro-/nanosystems for expanding their application range and improving their practical value.

**FIGURE 1:**
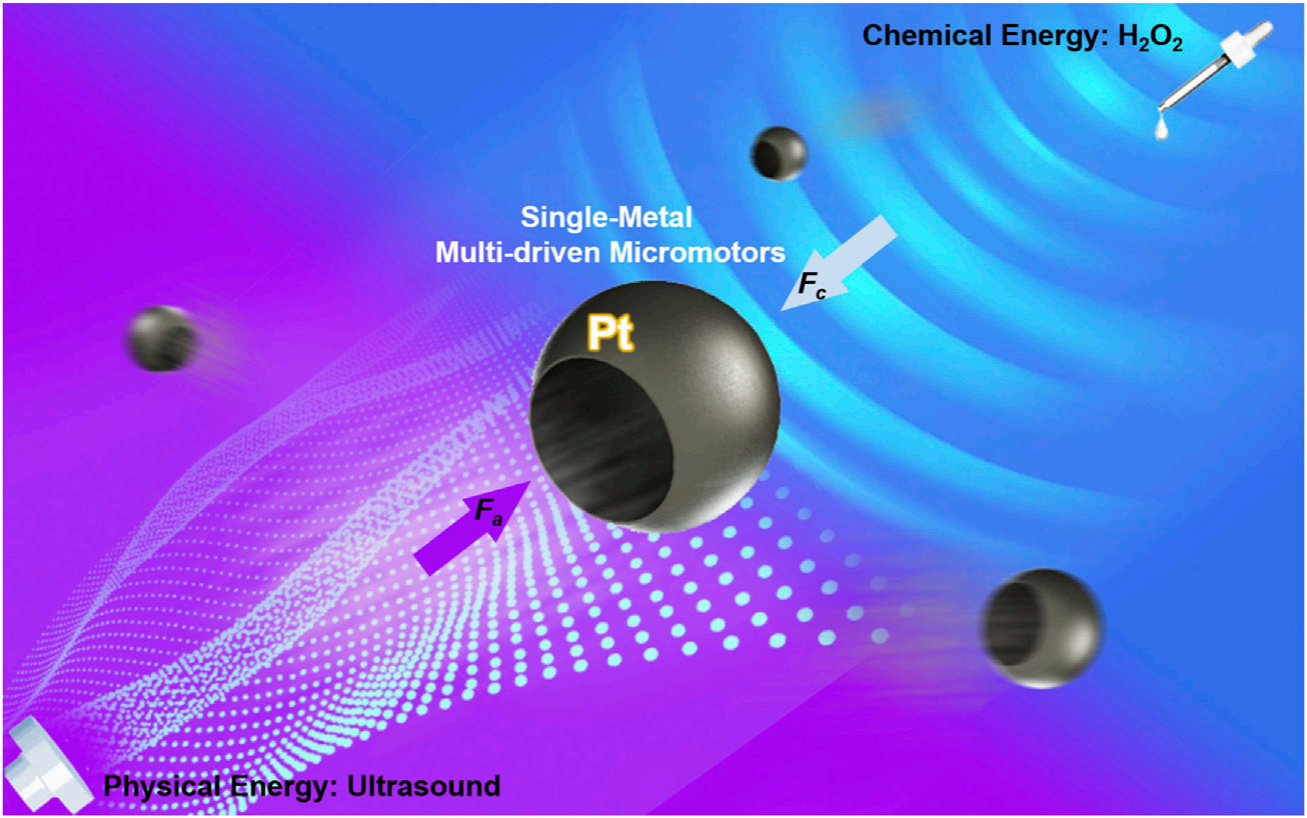
Schematic of hybrid Pt micromotors operating in a complicated environment, either with fuel or without fuel. *F*_*c*_ is the force generated by chemical reaction; *F*_*a*_ is the force generated by physical stimuli.

Here, we demonstrate an attractive design and present a dual-driven micromotor based on only one metal Pt, but also with easy fabrication—a very common sphere-template approach. The resulting Pt micromotors combined two driven modes (Figure 1): 1) in chemical fuel environment, they can be efficiently propelled toward their concave sides in the presence of H_2_O_2_ due to the chemical reaction between H_2_O_2_ and Pt; 2) in fuel-free environment, the micromotors can also be propelled efficiently toward their convex sides under acoustic fields due to the non-uniform acoustic energy distribution. In addition, the propulsion of such two-in-one micromotors can be flexibly and efficiently tuned by adjusting the fuel concentration or the operating voltage of the ultrasound transducer. Furthermore, these motors exhibit attractive group motion control, including aggregation group migration and dispersion. Such concise design with advanced hybrid operations could expand the scope of micromotor manipulation and provide an attractive route for achieving precise control of micromachines.

## Methods

### Synthesis of Pt Micromotors

The fabrication process is illustrated in Figure 1. Polystyrene beads (PS, 2 μm, Tianjing Beisile, Inc.) were first dispersed over a glass slide as the template. A platinum layer (150 nm) is deposited on the beads by the KYKY SBC-12 sputter coater. The obtained Janus microparticles were etched in CH_2_Cl_2_ for 1 h, and washed repeatedly with ethanol (Guangzhou Chemical Reagent Co.) and ultrapure water (18.2MΩ cm), three times each. Finally, we get the Pt shell micromotors.

### Speed Calibration Experiments

An aqueous H_2_O_2_ (Alfa Aesar #33323) solution was prepared and directly mixed with the motor droplets. The propulsion calibration experiments were performed by mixing 1.0 μl of the motor and hydrogen peroxide solutions each. The speed of the micromotors was also calibrated by using the software NIS-Elements Advanced Research 3.2. The “auto track” function in the software can track the marked objects frame by frame automatically and calibrate their velocities. We track at least 30 micromotors under fixed conditions, and then calculate the average velocities and the standard deviation.

### Equipment

The hybrid propulsion was carried out in a cell made in a covered glass slide (24 × 24 × 1 mm). A piezoelectric transducer (PZT) was attached to the bottom center of the glass slide to create the ultrasonic field. The continuous ultrasound sine wave was applied through the PZT, *via* a Tektronix AFG1062 arbitrary waveform generator, which was connected to a power amplifier (ATA 1200A, Aigtek). The continuous sine waveform and a voltage amplitude varied between 0 and 9 V, as needed for controlling the intensity of the ultrasonic wave. The chemically drive motion of the micromotors was recorded by using an inverted optical microscope (Nikon Instruments Inc. Ti-S/L100), coupled with a ×40 objective, using a Hamamatsu digital camera C11440 along with the NIS-Elements Advanced research 3.2 software. The acoustically driven motors are recorded by Olympus BX53 semi-motorized fluorescence upright microscope, coupled with a ×40 objective, using an Olympus DP74 camera. SEM images were obtained using a Tescan Maia 3 instrument, using an acceleration potential of 20 kV. The SEM images were obtained using fresh micromotor samples. Mapping elemental analysis was carried out using an Oxford EDX attached to the SEM instrument and operated by using Inca software.

### Numerical Simulation

The numerical simulation is finished by a commercial general-purpose simulation software COMSOL Multiphysics. In this simulation, the single metallic micromotor can be simplified to a hemispherical shell; the diameter and thickness are 2 μm and 150 nm, respectively. The CFD module is employed to calculate the distribution of oxygen, fluid flow, and resistance induced by the single metallic micromotor propulsion in low Reynolds number flow.

## Results and Discussion

**FIGURE 2:**
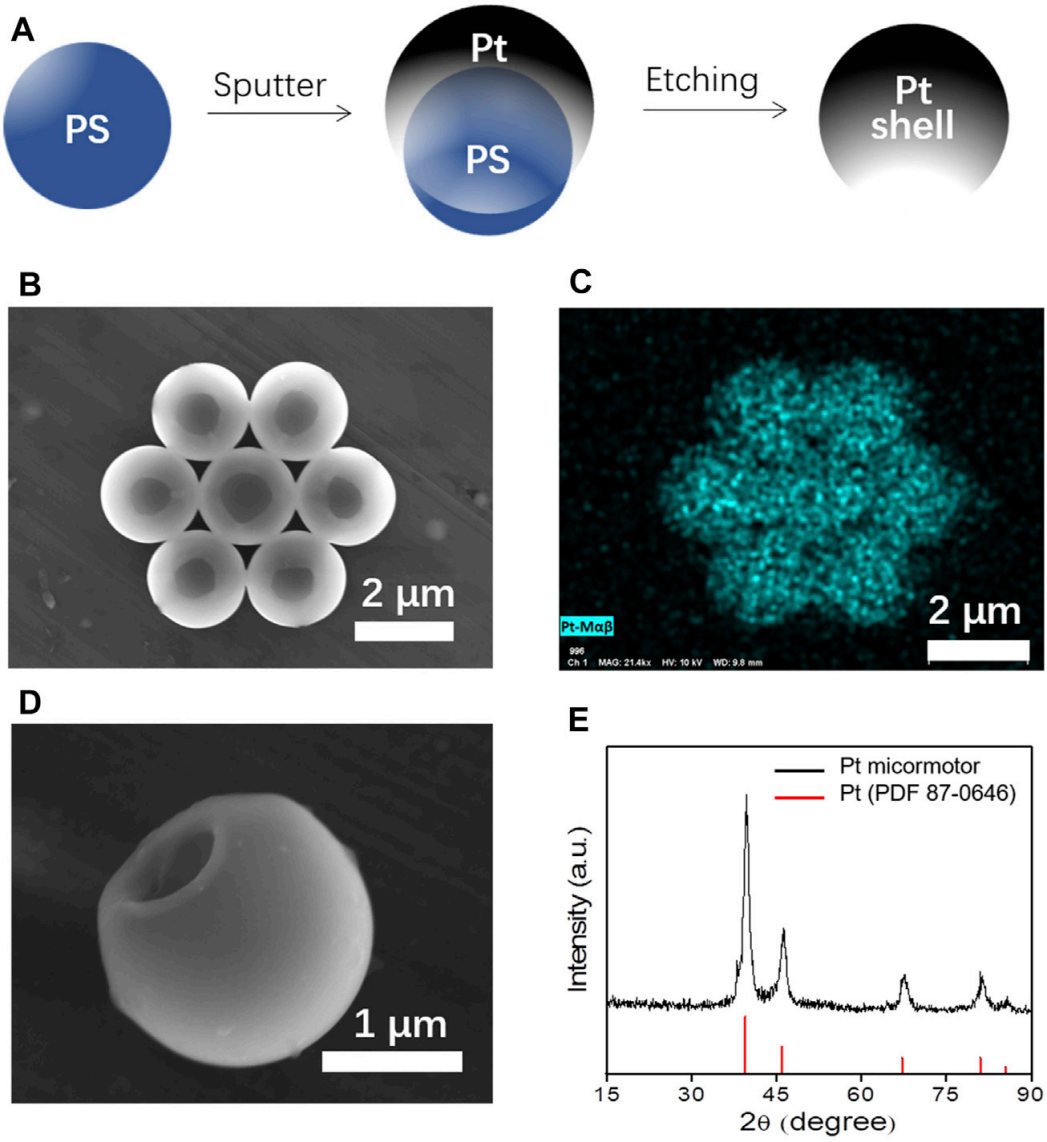
**(A)** Schematic of fabrication of Pt micromotor. **(B,C)** SEM of the Pt-based micromotors and the corresponding EDX results. **(D)** Magnified single Pt micromotor. **(E)** XRD results of the micromotors.

The new class of micromotors is constructed from a single element, Pt, where catalytic materials (essential for the chemical reactions) with concave structure (essential for the ultrasound actuations) are designed and fabricated (Figure 2A). The creation of such special architectures has been realized by the deposition of Pt on the PS microsphere (diameter 2 μm), followed by chemical etching. Based on the working principle of magnetron sputtering, the point between sphere and slide cannot be coated by any Pt; thus, a hole must be formed on the Pt shell. Another key point is that the thickness of the coating will gradually become thinner from the top center of the PS microsphere to the lower edge. As a result, when we removed the PS template by chemical etching, the thin edge will curl toward the side due to the stress and formed a hole like the SEM without sharp edge. The spherical structures with a hole (diameter is about 1 μm) are clearly characterized by scanning electron microscopy (SEM) (Figures 2B,D). Energy-dispersive X-ray spectroscopy (EDX) and X-ray diffraction (XRD) further confirmed such motors consisting of single element Pt (Figures 2C,E). The resulting microstructures offer an attractive chemically/physically propelled micromotor operation with the catalytic properties for the chemical propulsion and the concave structure for acoustic propulsion.

**FIGURE 3:**
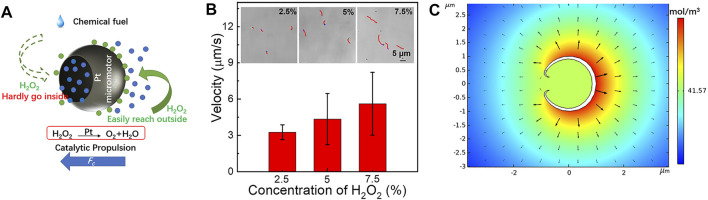
**(A)** Schematic of the chemical propulsions of the micromotor fueled by H_2_O_2_. **(B)** The influence of the fuel concentration on the speed of the micromotor s. The insets are track-lines of such motors fueled by the corresponding concentration of H_2_O_2_ in 3 s (taken from Supplementary Video S1). **(C)** Distribution of oxygen around the Pt micromotors.

Pt is a common catalyst which is efficient for H_2_O_2_ decomposition. The Pt surface catalyzes the decomposition of H_2_O_2_ to form oxygen gas (O_2_) and H_2_O as products (Figure 3A), and efficiently converts the chemical energy to the motors’ mechanical energy. Supplementary Video S1 in supporting information has clearly shown that such motors can be efficiently propelled in H_2_O_2_ solutions, and more interestingly, they move toward the concave side. That could be attributed to the special structure: microsphere with a hole. The only tiny hole greatly limits the H_2_O_2_ diffusion into the shell. H_2_O_2_ molecules are hard to reach and react with the inner side Pt. However, H_2_O_2_ molecules can easily contact the outer Pt surface and continuously react with it. As a result, the generated concentration gradient was formed around the motors and giving momentum to the fluid, leading to an osmotic gradient–induced force (*F*_*c*_) around motors, and further propelling the micromotor toward the concave side (Keh and Luo, 1995). Note that there were no bubbles observed. When the hydrogen peroxide concentration is higher than 7.5%, oxygen bubbles will be generated due to the excessively violent reaction, which will seriously affect the observation of motor motion behavior and the speed test. As a result, we think that when the concentration of hydrogen peroxide is lower than 7.5%, the reaction rate between Pt and hydrogen peroxide is not sufficient to make products form oxygen bubbles, but diffuse into the solution in the form of molecules, and form a concentration gradient around the motor, which then pushes the motor to move. Since the motors are driven by chemical reactions, the propulsion of the new single element Pt micromotors can be efficiently regulated by the adjusting the fuel concentrations due to the O_2_ gradient resulting from Pt-catalyzed decomposition of H_2_O_2_ (Figure 3B). The velocity of the motors can be increased from 3.26 to 5.61 μm/s with the improvement of the H_2_O_2_ concentration from 2.5 to 7.5%. The track-lines of Figure 3B inset and corresponding Supplementary Video S1 clearly reflect the attractive propulsion control of such single metal Pt micromotor. In order to confirm the diffusiophoretic propulsion mechanism, the locomotion mechanism of such single metallic micromotors in the presence of H_2_O_2_ is investigated by numerical simulations. It is clear to see that the O_2_ concentration at the outer side of the shell is higher than that at the inner side of the shell. These results further confirm that the single metal Pt micromotor is efficiently propelled by self-diffusiophoretic propulsion toward the concave side of the shell in the presence of fuel.

**FIGURE 4:**
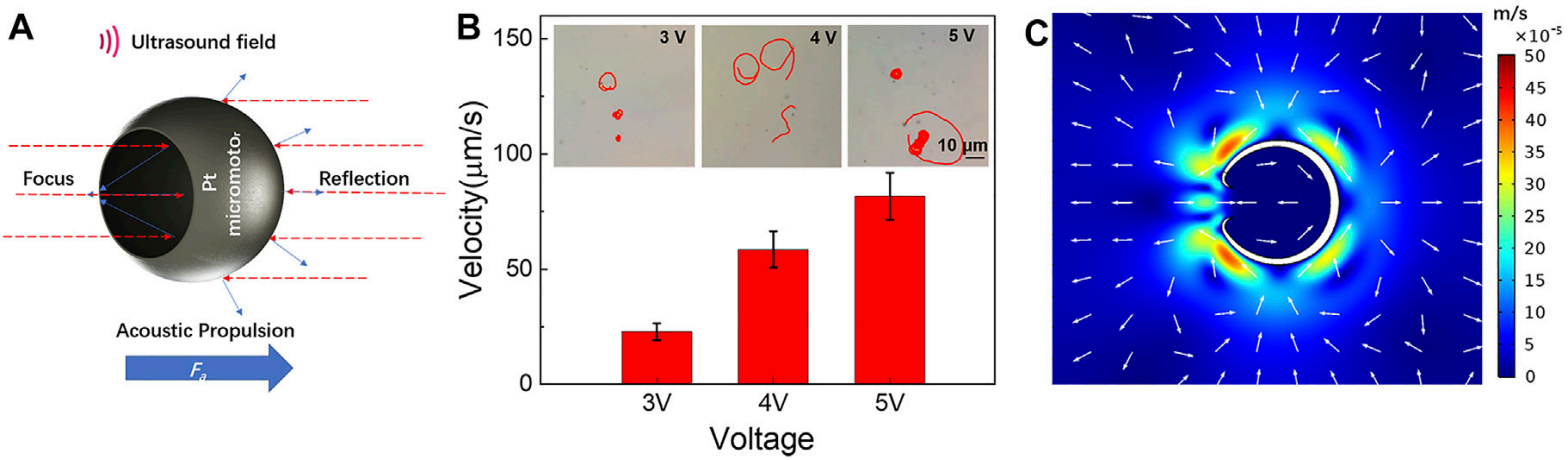
**(A)** Schematic of the acoustic propulsions of the micromotor under ultrasound stimulation. **(B)** The influence of the voltage of the 3 MHz ultrasound on the speed of the micromotors and track-lines of such motors under the corresponding voltage of 3 MHz ultrasound in 3 s (taken from Supplementary Video S2). **(C)** Acoustic streaming flow around the hemispherical shell in acoustic field.

Since our new hybrid nanomotor can be driven not only in solutions with chemical fuels but also be propelled in a non-fuel environment, once the fuel is exhausted, its speed could be easily regulated by changing the input parameters of the external field. In the absence of H_2_O_2_, by activating the acoustic mode, a micromotor can be viewed as a body oscillating in a uniform oscillating velocity field (Nadal and Lauga, 2014). Based on the physical mechanism of asymmetric steady fluid streaming, the inertial rectification of the time-periodic oscillating flow generates steady stresses on the nanomotor and forms an acoustic force (*F*_*a*_). In general, the force does not average to zero, resulting in a finite propulsion speed along the axis of the symmetry of the particle and perpendicular to the oscillation direction. The dimensional propulsion speed of the micromotor can be expressed as Eq. 1 (Nadal and Lauga, 2014)

$$v_{u}=\varepsilon R_{e}V^{⊥}v^{\left(1,1\right)},$$ (1)

where *ε* is the dimensionless small shape parameter, *R*_*e*_ is the Reynolds number, *v* ^(1,1)^ is the leading-order dimensionless propulsion speed, and *V* ^⊥^ is the relative amplitude of the particle oscillations that scales with the amplitude of the ultrasound field. Therefore, the speed of the hybrid micromotors levitated in solution under a fixed ultrasound wave frequency can be readily controlled by changing the amplitude of the driving voltage (Figure 4A). Accordingly, the propulsion performance of the hybrid micromotors can be efficiently regulated by changing the input parameters of the acoustic modes with the frequency of 3 MHz. As expected from Eq. 1, the speed increases from 22.84 to 58.52 and 81.62 μm/s on increasing the applied voltage with 3 to 4 and 5 V, respectively (Figure 4B). The inset of Figure 4B displays track-lines of the nanomotor over 3 s periods (corresponding to Supplementary Video S2) using diﬀerent applied voltages of the acoustic mode. The acoustic propulsion mechanism is based on the second-order acoustic streaming flow due to the oscillations of the sharp edges (Kaynak et al., 2016). Figure 4C shows the flow field of the single metal micromotors driven by oscillating of a sharp edge in an acoustic field. Similar phenomena are observed in other micromotors with a bow-like structure propelled in an acoustic field (Soto et al., 2016; Tang et al., 2019).

**FIGURE 5:**
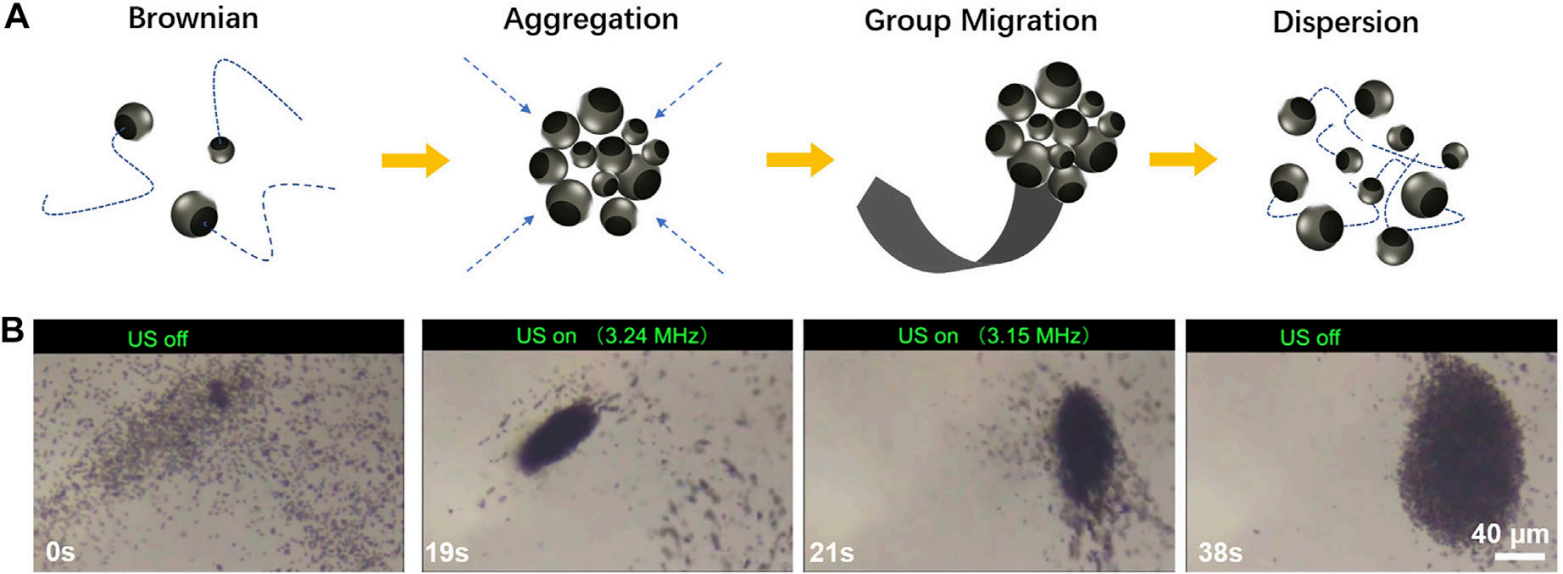
**(A)** Schematic of group motion behavior of the hybrid micromotors. **(B)** The time lapse images of the micromotors group motion control (taken from Supplementary Video S3).

In addition to the excellent catalytic propulsion and acoustic propulsion of a single motor, such motors also exhibit attractive group behavior (Figures 5A,B). Initially, motors in water usually exhibit Brownian motion. Once the acoustic field with 3.24 MHz and 3 V with a power amplifier was turned on, the motor aggregated immediately and formed a group with high density due to the acoustically generated pressure gradients. There are two waves generated in the device: one wave is generated by the transducer, and the other one reflected by the cover slide, both of them with equal amplitudes and wavelengths. The interference between the two waves produces a standing wave, which leads to the formation of nodes and antinodes, and results in pressure gradients, which can drive the motors in solution toward low-pressure regions (Woodside et al., 1997). Furthermore, when the frequency of the acoustic field changed from 3.24 to 3.15 MHz, the group of the motors can be move together to a new position. Frequency change leads to wavelength shift, which results in changes in the location of the pressure nodes and further drives the migration of the swarm toward a new location (Ding et al., 2012). Then, upon removal of the acoustic field, the Brownian motion dominates the individual motors motion again, which drives the motor swarm to disperse quickly. The attractive control of group behavior offers considerable promise for various applications range from nanomedicine to cargo transport.

## Conclusion

In conclusion, we have reported a novel design for a dual-driven mode micromotor which is fabricated by only one metal, Pt, with a special structure: spherical shell with a hole. The resulting simple structure can be easily and efficiently realized by the traditional sphere-template method. In addition, such motors can be efficiently propelled by two driven modes. The motors can harvest the propelling forces by utilizing the catalytic H_2_O_2_ decomposition over Pt for efficient chemical propulsion in fueled environments, as well as utilize acoustic forces from the acoustic field for propulsion in fuel free environments. Advantageously, these new single metal Pt micromotors also exhibit an excellent group behavior, including aggregation, group migration, and dispersion. Such novel micromotors we propose here hold considerable promise for designing simple but smart “robots” that autonomously reconfigure their propulsion in response to changes in their surrounding environment, which are expected to advance the field of designing specialized artificial nanomachines.

## Data Availability

The original contributions presented in the study are included in the article/Supplementary Material; further inquiries can be directed to the corresponding authors.
